# Emerging approaches to investigating functional protein dynamics in modular redox enzymes: Nitric oxide synthase as a model system

**DOI:** 10.1016/j.jbc.2025.108282

**Published:** 2025-02-08

**Authors:** Ting Jiang, Megan C. Thielges, Changjian Feng

**Affiliations:** 1 College of Pharmacy, University of New Mexico, Albuquerque, New Mexico, USA; 2 Department of Chemistry, Indiana University, Bloomington, Indiana, USA

**Keywords:** nitric oxide synthase, calmodulin, conformational change, electron transfer, flavoprotein, protein dynamics, protein complex, mass spectrometry, infrared spectroscopy, electron paramagnetic resonance

## Abstract

Approximately 80% of eukaryotic and 65% of prokaryotic proteins are composed of multiple folding units (*i.e.*, domains) connected by flexible linkers. These dynamic protein architectures enable diverse, essential functions such as electron transfer, respiration, and biosynthesis. This review critically assesses recent advancements in methods for studying protein dynamics, with a particular focus on modular, multidomain nitric oxide synthase (NOS) enzymes. Moving beyond traditional static "snapshots" of protein structures, current research emphasizes the dynamic nature of proteins, viewing them as flexible architectures modulated by conformational changes and interactions. In this context, the review discusses key developments in the integration of quantitative crosslinking mass spectrometry (qXL MS) with AlphaFold 2 predictions, which provides a powerful approach to disentangling NOS structural dynamics and understanding their modulation by external regulatory cues. Additionally, advances in site-specific infrared (IR) spectroscopy offer exciting potential in providing rich details about the conformational dynamics of NOSs in docked states. Moreover, optimization of genetic code expansion machinery enables the generation of genuine phosphorylated NOS enzymes, paving the way for detailed biophysical and functional analyses of phosphorylation's role in shaping NOS activity and structural flexibility; notably, this approach also empowers site-specific IR probe labeling with cyano groups. By embracing and leveraging AI-driven tools like AlphaFold 2 for structural and conformational modeling, alongside solution-based biophysical methods such as qXL MS and site-specific IR spectroscopy, researchers will gain integrative insights into functional protein dynamics. Collectively, these breakthroughs highlight the transformative potential of modern approaches in driving fundamental biological chemistry research.

### Multidomain redox proteins and conformational dynamics

In this review, we will focus on recent advances made since 2019 in nitric oxide synthase (NOS), a family of oxidoreductases, as a model system to highlight method developments in characterization of functional protein dynamics in modular redox enzymes. To begin with, electron transfer (ET) in biology drives the processes of living systems. From cellular respiration to photosynthesis, metabolism and molecular signaling, ET within and between proteins is a fundamental biological process of immense importance to living organisms.

Proteins are inherently dynamic biomolecules. Approximately 80% of eukaryotic proteins, and nearly two-thirds of their prokaryotic counterparts comprise multiple domains connected by flexible linkers (1, 2). These dynamic protein architectures, enabled by linker regions, underpin diverse, essential functions, including ET. Despite their functional importance, studying large multidomain proteins experimentally remains challenging. Protein motions can take place on a wide range of timescales: large-scale motions, such as domain rearrangements, typically occur over the millisecond to second timescale, while localized motions, including bond vibrations and side chain rotations, take place on much faster timescales, ranging from femtoseconds to nanoseconds. Large-scale motions, for instance, are necessary to facilitate ET over long distances between redox centers in redox proteins, since through-space ET is limited to below 20 Å. This is to position the electron donor and acceptor domains closer to each other and at an optimal relative orientation. Once redox centers are brought into proximity, small-scale motions and interactions, which determine the local flexibility, are important for understanding the mechanisms governing ET between them. Partner domains bind in dynamic complexes that enable population of various conformations, only a subset of which may allow for efficient ET (3). Dynamic complexes, formed by flexible interactions among protein domains that adopt various conformations, are essential for balancing optimal ET with efficient enzyme turnover. Therefore, understanding protein function necessitates a shift from static structure-function paradigms to incorporating biomolecular dynamics.

In exploring how Nature has optimized protein dynamics for ET, it is essential to recognize that biological ET differs from that between small molecules. Biological ET occurs between redox centers embedded within protein environments that are structurally heterogeneous and undergo motions over a broad range of magnitudes and timescales. These motions are shaped by the conformational energy landscape, *i.e.*, the dependence of free energy on protein conformation. The energy landscape, describing the distribution of conformational states and their dynamics, can be modified by partner protein binding and/or post-translational modifications (PTMs), serving as a mechanism for regulation of biological processes. This dynamic nature is fundamental to enabling biological function and is widespread in biology, given the modular assembly and flexible nature of biological ET systems (4). It is the remodeling of these energy landscapes that controls biological function and opens up new opportunities for therapeutic intervention. While the concept of landscapes is central to biological function, experimental and computational tools to interrogate the spatial and temporal properties remain limited. Furthermore, linking motions and conformations to mechanisms requires integration of multiple, complementary experimental, and computational techniques (5). Notably, solution-based biophysical methods are crucial for studying functional protein dynamics as they preserve native conformations and enable analysis of protein interactions, conformational changes, and stability in physiologically relevant environments — some even offering real-time monitoring. These molecular biophysics methods provide rich valuable insights into protein behavior, including how different domains within a protein and/or between proteins interact and move. Given these challenges and opportunities, there is growing interest in defining the interplay between dynamics and ET in multidomain redox proteins.

### Why is NOS a paradigmatic model system for studying multidomain protein dynamics?

In this context, NOS stands out as a paradigmatic model system for studying the dynamics of multidomain redox proteins. Mammalian NOS is responsible for producing nitric oxide (NO), an intercellular signaling molecule critical for neurotransmission, vasodilation, and innate immune response. For example, NO is uniquely well-suited for local neurotransmission because it is a short-lived diffusible gas. Unlike classical neurotransmitters, NO cannot be stored in vesicles. As a fairly reactive free radical, NO production by NOS is tightly regulated by multiple factors (6). NOS is subject to intricate regulatory mechanisms that are common in multidomain proteins, including control *via* protein-protein interactions (PPIs), PTMs such as phosphorylations, regulatory regions, and ligand binding. Moreover, the versatile functions of NOS isoforms depend on various splice variants (7) and regulation of protein expression (8). Such complex interplay of multiple regulatory factors and layers underpin proper NO biosynthesis by the NOS isoforms, driven by various biological stimuli, where electron transport across domains is essential.

#### NOS domains and interdomain electron transfer

Mammalian NOS enzymes function as monooxygenases, producing NO and citrulline from l-arginine (l-Arg), NADPH and O_2_:

2 l-Arg + 3 NADPH + 3 H^+^ + 4 O_2_ → 3 NADP^+^ + 2 Citrulline + 2 NO + 3 H_2_O

Eukaryotic NOS evolved through a series of gene fusion events, giving rise to a modular enzyme that contains both heme and flavin domains. Hallmark features of NOSs include multidomain architectures with highly flexible linkers, allowing for dynamic, regulated interdomain electron transfer (IET). Mammals possess three NOS isoforms: neuronal, endothelial, and inducible NOS (nNOS, eNOS, and iNOS, respectively). Regardless of the isoform, NOS is a large homodimeric enzyme, with each subunit composed of an N-terminal heme-containing dimeric oxygenase domain, a C-terminal reductase domain composed of FMN- and FAD-containing subdomains, and a calmodulin (CaM)-binding linker in between (Fig. 1*A*). During catalysis, electrons are transported from the reductase domain to the catalytic heme domain (NADPH → FAD → FMN → heme), where the l-Arg substrate is oxidized to produce NO. A Ca^2+^-sensing protein, CaM, activates NOS by binding to the linker region connecting these two domains (9). Furthermore, PTMs such as phosphorylation regulate the biosynthesis of NO *in vivo*. Despite the lack of a crystal structure of full-length NOS, single-particle electron microscopy (EM) studies (10, 11) revealed its general architecture, showing that while the domains remain relatively rigid, they can associate in different configurations through flexible linker regions. NOS function relies on its highly flexible architecture (11, 12).

**Figure 1:**
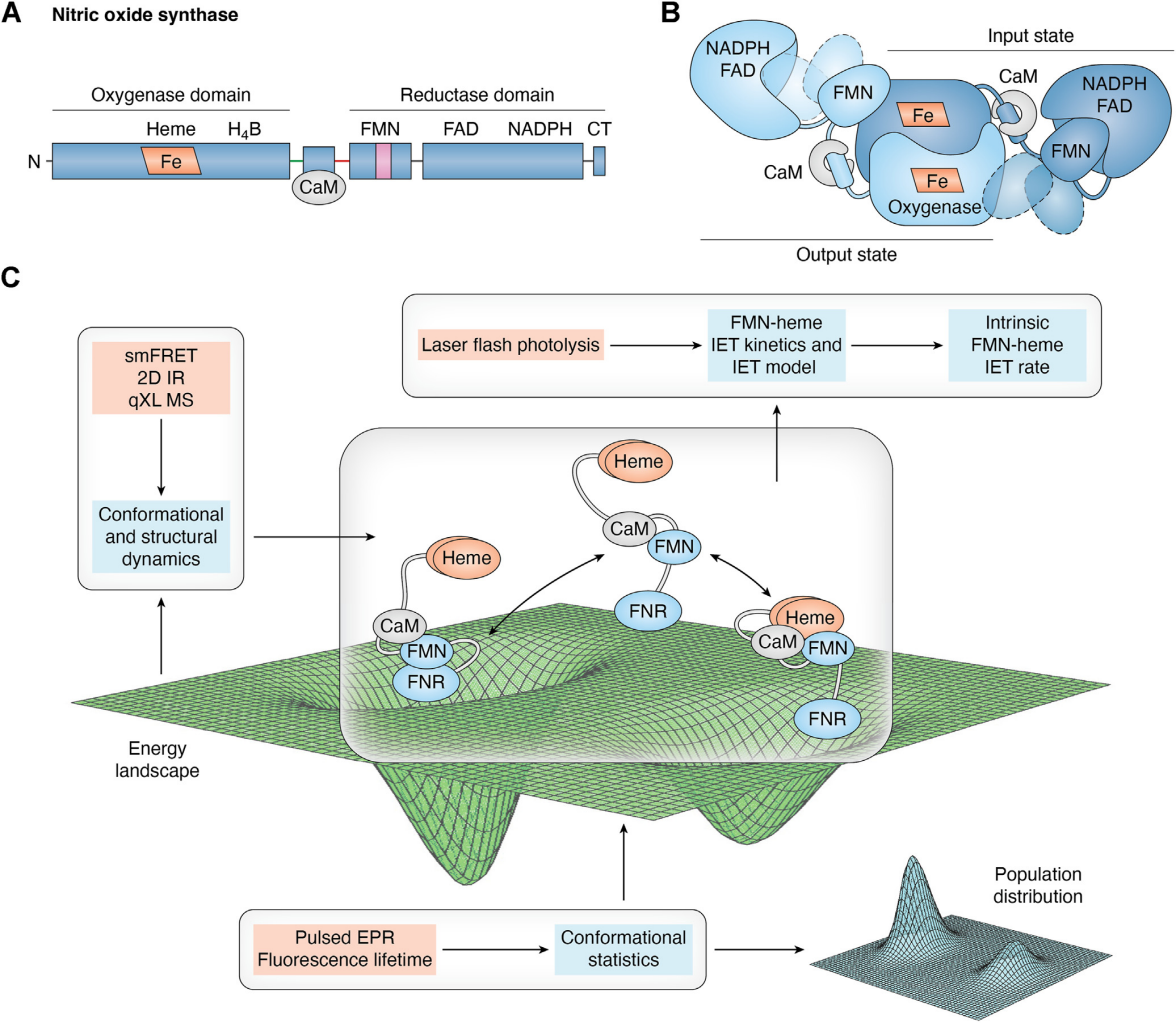
**Structural organization, conformational dynamics, and biophysical characterization of mammlian NOS isoforms.***A*, the NOS isoforms are composed of an N-terminal heme-containing oxygenase domain and a C-terminal reductase domain, connected by a linker region. This linker contains a canonical CaM-binding site and hinge regions connected to the heme and FMN domains. The dimeric oxygenase domain encloses the catalytic heme center and the binding sites for (6R)-5,6,7,8-tetrahydrobiopterin (H_4_B) cofactor and l-Arg substrate, while the reductase domain includes a mobile FMN subdomain, and an NADPH/FAD subdomain (also referred to as ferredoxin-NADPH reductase (FNR) subdomain); the prefix “sub” is frequently omitted. Notably, the nNOS and eNOS isoforms possess an autoregulatory (AR) insert (*magenta*) within the FMN subdomain, which is absent in iNOS. The C-terminal tail (CT) varies in both length and sequence across the three NOS isoforms. *B*, the NOS function requires conformational changes enabled by the CaM binding to the linker: it unlocks the FMN domain from the electron-accepting “input state” and allows large-scale motions of the FMN subdomain to bring the FMN and heme domains together, forming the electron-donating “output state” (27). The tethered FMN subdomain shuttles between these two types of docking positions through free, undocked states (dashed structures). Furthermore, local adjustment/sampling continues in the docking complex (15). The lack of high-resolution structures of full-length NOSs obscures the molecular basis for conformational dynamics induced by external cues such as CaM binding to NOS. *C*, selected current in-solution biophysical techniques for deciphering the NOS functional protein dynamics. The experimental methods are indicated in the blush-colored text boxes.

Of note is that the intersubunit FMN−heme IET is essential for the delivery of electrons required to activate O_2_ and the subsequent substrate oxidation and NO production in the heme domain (13). The rates of the catalytic reactions at the heme site are generally comparable to that of the IET (14, 15). The FMN−heme IET thus plays a key role in NOS function, which warrants detailed investigation of the conformational aspects that determine its efficiency (16). Notably, a laser flash photolysis approach enables precise determination of kinetics of the discrete FMN−heme IET step in NOSs (17, 18), providing significantly greater insight into structure-function relationships than steady-state kinetic analysis that cannot resolve this specific IET process. In contrast to the stopped-flow method used in other studies, this CO photolysis technique (17, 18) uniquely allows for direct monitoring of FMN–heme IET, making it a valuable tool for dissecting this critical mechanistic step. Moreover, the IET rate serves as a measure for the formation of the ET-competent interdomain docking state. This method proves particularly powerful when combined with site-specific mutagenesis, enabling elucidation of key, specific structural determinants underlying the ET processes in the NOS isoforms (19, 20, 21, 22, 23, 24).

#### Conformational dynamics and regulation of NOS

The NOS enzyme produces NO through tightly regulated ET processes (25). During catalysis, NOS facilitates an intricate sequence of IET steps to shuttle the NADPH-derived electrons from the reductase domain to the oxygenase domain. Because through-space ET is efficient only over distances of a few angstroms, NOS function requires sequential conformational changes, which reposition the tethered FMN domain with respect to the FAD and heme domains so that the FAD−FMN and FMN−heme IETs can occur efficiently in the input and output states, respectively (Fig. 1*B*). In a catalytic cycle, the NOS domains move within the limits determined by the length and flexibility of the tethers and form docking complexes. Such tethered shuttle model was originally proposed by Salerno in 2003 (26) and supported by extensive kinetics (27), structural (10, 11), and spectroscopic (9, 28) data. The ancestral ET systems, such as flavodoxin/flavodoxin reductase, function with flavodoxin serving as a shuttle between the two-domain reductase and other components. In NOS, a modular enzyme that incorporates domains derived from these ancestral systems, a tethered shuttle still operates. Here, the tethers correspond to the linkers between the FMN-heme and FAD-FMN domains. The unique feature of NOS, however, is its regulation by the partnering protein CaM. Several CaM-responsive control elements/regions in NOS isoforms, such as the autoregulatory (AR) insert within the FMN domain of nNOS and eNOS and the C-terminal tail (CT) (Fig. 1*A*), synergistically modulate NO production activities (29); these regulatory elements are NOS-unique and, for example, do not exist in the homologous P450 reductase proteins.

It is important to emphasize the differences between mammalian NOS and P450 protein families, as these distinctions are essential for understanding the unique regulatory mechanisms of NO biosynthesis. Although NOS is often referred to as a P450-like protein due to both being heme-thiolate proteins with similar yet distinct reductase partners (as mentioned in the previous paragraph), there are key differences in their regulation mechanisms. NOS relies on H_4_B as a crucial electron donor to stabilize the oxyferrous complex, preventing its conversion to ferric heme and the formation of superoxide. Additionally, while P450 enzymes require a separate ET reductase protein and inter-protein binding, NOS integrates both ET-partnering domains into a single polypeptide chain, keeping them tethered. Moreover, in P450 enzymes, substrate binding induces dramatic conformational changes in the binding pocket, allowing for broader substrate selectivity. In contrast, the crystal structure of the NOS heme domain remains largely unchanged upon l-Arg binding. Furthermore, while substrate binding in P450 enzymes alters the spin state of the heme iron, thereby triggering enzymatic activity, NOS activation is primarily mediated by CaM binding. The functional dynamics in NOS are governed by interdomain conformational changes, rather than alterations within the heme domain as seen in P450 enzymes.

Emerging evidence suggests that the functional differences among various NOS proteins are due to variations in the interaction energies between the NOS domains as well as with CaM (30). These energies are dependent on both distance and orientation, and this dependence represents a multidimensional energy landscape (Fig. 1*C*); the free energy landscape represents enzyme structures as a multidimensional energy surface with 'hills' and 'valleys' separating energy minima. Recent advances in experimental techniques have provided powerful tools for studying NOS isoforms (Fig. 1*C*)—some of which were inaccessible just a few years ago (11, 30, 31, 32, 33, 34). These techniques include pulsed EPR (28, 35), hydrogen-deuterium exchange mass spectrometry (HDX MS) (13, 36), single particle EM (10, 11, 37), and single-molecule fluorescence resonance energy transfer (9, 38). For example, a key strength of the HDX MS technique is its ability to assign structural rearrangements to specific residues, making it particularly useful for mapping the structural consequences of CaM- or ligand-binding to NOS. This data-rich approach provides valuable insights into the molecular dynamics of the NOS enzyme (13, 36). Another noteworthy approach in measuring distance and distance distribution between electron spins in proteins is pulsed EPR, which encompasses double electron-electron resonance (DEER) (39) and relaxation-induced dipolar modulation enhancement (RIDME) (40) techniques. DEER, also referred to as pulsed electron-electron double resonance (ELDOR), is optimal for studying pair of the FMN and FAD semiquinone radicals (35), while RIDME is uniquely suited for measuring the distances in pair where one or both spins are paramagnetic metal ions, such as the low spin ferric heme center present in NOS (40). These two pulsed EPR methods were also applied in distance measurements of other modular protein systems (40, 41).

These recent elegant studies have provided valuable insights into NOS structural dynamics, particularly the large-scale domain shuttling motion. It is now known that NOSs contain several structural elements that regulate ET both within and out of its reductase domain. Some of the regulatory elements are common to and present in related di-flavin reductases (4), while others are unique to the NOS enzymes, including a CT that is present in all three mammalian NOSs but differs in both its length and sequence, and an AR insert that is located in the FMN subdomains of eNOS and nNOS but is absent in iNOS (27). These control elements suppress ET in the reductase domain without CaM (42), while CaM binding alleviates the suppression under Ca^2+^-repleted conditions. Both CaM and these common and unique structural elements putatively underpin distinct activities of NOSs by biasing their conformational landscapes (43).

Despite these advancements, several key aspects remain poorly understood: How are the differences in the regulation of the NOS isoforms related to specific, dynamic elements/regions? How do PPIs affect functional conformations and dynamics? How do such PPIs and PTMs regulate the NOS conformational behavior and function at the molecular level? These are also fundamentally important questions on multidomain proteins in general, as both conformational dynamics and mechanisms of regulation remain largely unclear. Integrative biophysical approaches are necessary to deconvolute functional protein dynamics because each method has specific limitations, and no single approach offers a comprehensive description. For example, single particle EM has provided structural snapshots of the NOS conformational states (10, 11). However, it cannot quantitatively describe the conformational dynamics or statistics. Moreover, single particle EM could only provide rather low resolution (60−74 Å) structural information on the molecular architecture of the NOS holoproteins (11). More recently, the nNOS and nNOS-CaM complexes were examined by cryo-EM (31). The maps definitively underscore the conformational dynamics. The dimeric oxygenase domain structure has been resolved to approximately 4 Å, revealing atomic-level detail, but the reductase domain remains unresolved at high resolution (31). This continues to emphasize how intransigent NOS has been to EM studies. Cryo-EM is being developed rapidly, and issues with atomic resolution and freezing out of complexes are expected to be solved soon. Applicability of NMR to studying the NOS protein dynamics has been hindered by the large size of the NOS holoenzymes (∼240–320 kDa) and by the fact that one needs to study the relative motion of large, relatively rigid modules connected by long random coil tethers, *i.e.*, under the conditions where the interactions between the magnetic nuclei from different modules are minimal and the system exists as a dynamic ensemble.

The NOS enzyme has been investigated for decades and has proven quite difficult because this multidomain system is large and intrinsically complex, involving not only conformational changes, but also PPIs and PTMs. A central question is how the functional domains collaborate to respond actively to external cues, such as CaM-binding and phosphorylations, which add layers of regulation to biological processes. Several seminal reviews on NOS enzymology, regulation mechanisms (25, 27, 43, 44, 45, 46), and chemical pathways (47) have been published between 2001 and 2019. Noteworthy reviews on in-solution kinetic and spectroscopic methodologies (16, 48, 49, 50) also offer valuable insights − in-solution methods are crucial for capturing the native, dynamic behavior of proteins, and their interactions in a physiologically relevant environment. In the following sections, we will explore mechanistic questions and highlight recent advances since 2019 in emerging experimental approaches that are beginning to be used to tackle these mechanistic questions and technical issues. Additionally, we will critically assess the advantages and limitations of the experimental and computational methods. Finally, we will share our perspective on future directions in the NOS enzymology and the studies of other modular redox enzymes.

### Investigating NOS conformational dynamics by quantitative cross-linking mass spectrometry

#### Exploring protein dynamics and interactions through XL MS

In addition to the CaM-heme(NOS) domain-domain docking (13, 28), the inter-protein CaM-NOS interactions are modulated by the AR insert within the FMN subdomain and the CT (Fig. 1). These control elements are disordered—only a small helical portion in the AR insert and half of the CT are observed in the crystal structure (51); they are also NOS-unique regulatory elements (42). In the absence of CaM, these control elements (AR insert and CT), along with NADP^+^ binding, suppress ET across the NOS domains. CaM binding relieves the inhibitory factors to promote the electron transport required for NO production. Furthermore, the AR insert is proposed to facilitate the formation of the FMN-heme docking complex (52). Besides AR insert and CT, two flexible tethers connect the FMN subdomain to the other NOS domains (Fig. 1*A*) and hypothetically tune interactions with partnering domains for ET and catalysis (30, 53); these tethers exhibit varied compositions and lengths among nNOS, eNOS, and iNOS isoforms. The NOSs thus offer an exemplary model to study the role of flexible motifs in regulating modular proteins. Yet, the exact interplay between these regulatory elements and CaM-NOS interactions remains elusive due to their inherent flexibilities.

The dynamic behavior, specifically the relative positioning of these control elements, is highly intriguing but cannot be resolved by cryo-EM or X-ray crystallography. The cross-linking mass spectrometry (XL MS) approach can identify amino acid residues in spatial proximity, providing medium-resolution information on relative domain positions and orientations without imposing structural rigidity biases (54, 55, 56). XL MS works by chemically linking protein residues that are close in space; after digestion, these cross-linked residues are identified using LC-MS/MS, revealing their original proximity. The maximum effective length of the cross-linking reagent serves as a distance constraint (57, 58). By elucidating the spatial proximity of amino acid residues, XL MS provides extensive distance restraints for the protein and protein complexes, surpassing the capabilities of traditional techniques (59). In the context of NOS proteins, XL MS offers an effective approach to examine the highly dynamic CaM-free state, which poses challenges for crystallography and high resolution cryo-EM. Moreover, the regulatory regions of NOS, such as the AR insert and CT, are ripe targets for lysine-based crosslinking.

Furthermone, in recent years, quantitative XL MS (qXL MS) methods have emerged as a powerful tool for studying the dynamic interactions and conformations of protein complexes under varying physiological conditions (60, 61, 62). Changes in the relative abundances of specific cross-linked peptides reflect alterations in protein/domain interactions and conformations. Although successful, the reproducibility of measuring low-abundance cross-links remains a challenge. To address this, targeted qXL MS is crucial to enable precise quantitative measurements, enhancing sensitivity and reliability in assessing these crosslinks (62). We have thus refined a parallel reaction monitoring (PRM)-based targeted qXL MS protocol for NOS studies. By analyzing a large number of transitions (*e.g.*, 10) per MS^1^ peak and a sufficient number of data points (*e.g.*, ≥8) across each LC peak, PRM provides highly specific and sensitive quantitation of identified cross-links, further bolstering confidence. This targeted approach allows label-free comparative analysis of selected cross-links across multiple samples, empowering detailed comparison. Beyond NOS, this qXL MS approach is broadly applicable for studying other multidomain proteins and protein-protein complex systems.

XL MS provides distance constraints, but the application of XL MS in protein science remains underexplored, partly due to the scarcity of high-confidence experimental or computational structural models to structurally interpret the MS data. Recent developments in computational tools harnessing artificial intelligence, *e.g.*, AlphaFold (63), have revolutionized protein research by enabling accurate modeling of protein domains and complex structures. These tools have been widely embraced by the research community as a transformational breakthrough. Despite their success, current structure prediction methods, such as Rosetta (64), ITASSER (65), and AlphaFold 2 (66), are primarily optimized for single-domain proteins. Predicting relative domain-domain positioning often remains of relatively low confidence due to the complexity of domain-domain interactions and conformational changes within a multidomain protein. AlphaFold-Multimer, for instance, relies heavily on known structures in protein databases to infer and predict the folding patterns, which limits its ability to accurately predict structural variations arising from interactions between multiple domains. To bridge this gap, an emerging approach combines actual experimental data, such as NMR (67) and XL MS (68), with computational modeling. This enhances the accuracy of multidomain protein structure predictions by providing additional constraints and proximity context, tackling the inherent challenges of modeling complex domain interactions.

To demonstrate the power of this integrative approach, we next present two case studies that combine experimental XL MS with AlphaFold 2 computational modeling. These studies investigate protein dynamics in monomeric nNOS reductase (nNOSred) (32) and dimeric oxygenase/FMN (oxyFMN) (69) proteins, both of which are valid models for exploring ET mechanisms in NOS isoforms.

#### Probing structural changes in a monomeric nNOS protein

In our initial efforts to establish the XL MS workflow (32), we selected a rat nNOSred construct containing residues 695 to 1429, which includes the NADPH, FAD, and FMN subdomains, as well as the CaM-binding linker (70). The nNOSred construct is monomeric in solution but fully active in supporting ET across its domains. Notably, the monomeric nature of nNOSred is advantageous for XL MS, as it eliminates the challenge of distinguishing between inter- and intra-subunit cross-links.

MS-cleavable crosslinkers, such as disuccinimidyl dibutyric acid (DSBU) (71, 72, 73), are used to facilitate identifying genuine cross-linked peptides while reducing false positives from non-specific attachments (71, 72). Crosslinking data of the nNOSred-CaM complex revealed interactions between the AR and β-finger regions and CaM sites, corroborating previous HDX MS data comparing nNOS samples in the presence and absence of CaM (36). The β-finger and AR had been proposed to be near the bound CaM (74) and interact with CaM (75), respectively, but such model lacked experimental confirmation. The XL MS data provide the first direct experimental evidence of their close proximity, based on distance range information. It is important to note that crosslinking introduces a unique dimension of data—distance restraints—that HDX MS cannot technically distinguish. Additionally, recent work using XL MS successfully captured transient, dynamic CaM-nNOS conformations that eluded detection by HDX MS (31). While this marks an exciting advancement, it is worth mentioning that the study was qualitative in nature (31).

To evaluate functionally relevant condition-dependent structural changes, PRM-based targeted qXL MS was employed to compare the relative abundances of key intra- and inter-protein cross-links in the presence or absence of NADP^+^ ligand and partnering protein CaM (32). All unique crosslinks were compiled into a PRM inclusion list for targeted LC-MS runs, with peptide intensities normalized across each run using Skyline (76). Notably, NADP^+^ plays a physiologically relevant regulatory role (77). Without CaM, NADP(H) binding is believed to lock the nNOS reductase domain in a conformation that restricts the motion of the FMN subdomain (51); this “conformational lock” is thought to be released upon CaM binding (77).

The qXL MS data revealed conformational differences among nNOSred alone, nNOSred with NADP^+^, nNOSred-CaM, and nNOSred-CaM with NADP^+^ samples. Distinct effects of CaM and NADP^+^ on the cross-linking patterns in nNOSred were observed (32). CaM binding to nNOSred induces significant global conformational changes, whereas NADP^+^ primarily affects the crosslinks in the NADPH-binding subdomain. Additionally, CaM increases the abundance of intra-nNOS cross-links, which are associated with the formation of related inter-CaM-nNOS cross-links. These XL MS results demonstrate that both CaM and NADP^+^ site-specifically modulate the conformational landscape of nNOSred.

#### Mapping intersubunit FMN-heme interactions in a dimeric nNOS construct

The XL MS workflow for the monomeric nNOSred construct requires further refinement to accommodate more complex systems, such as homodimeric NOS proteins. While the monomeric nNOSred construct is a useful model for electron transport within the reductase domain, in NOS holoenzymes, electron shuttling continues across homodimeric subunits, which is essential for completing the NOS catalytic cycle. Oligomeric proteins, in general, consist of multiple identical domains that assemble and interact to facilitate dynamic functionality (78). A particular challenge in studying homo-oligomeric proteins by XL MS is the need to differentiate between inter- and intra-subunit cross-links (79). To address this, analyzing cross-linked monomeric *versus* dimeric bands were used in early literature to distinguish between inter- and intra-subunit crosslinks (80, 81). But there are limitations: monomer bands may not always mirror the intra-monomer crosslinks in the dimer band (81). Isotopically labeling protein for monomer-specific identification before dimerization (79) is not feasible for NOS either, as its dimerization is essential for proper folding and co-factor incorporation.

To address this complexity, we have extended our approach to examine a homodimeric nNOS oxyFMN construct (69), a well-established model for the NOS output state (17, 18); see also Figure 3*E* below for its design. Another consideration is the availability of structural models: we can obtain reasonable AlphaFold 2 structural models for the oxyFMN construct, which is necessary for mapping and assigning the observed crosslinks in the context of domain-domain interactions. Notably, our top AlphaFold 2 predictions give trustworthy models for the nNOS oxyFMN-CaM complex, which have been validated against both the crystal structures of individual domains and the inter-subunit docking interface (13): the heme active site and H_4_B cofactor binding site align with the nNOS heme domain crystal structure, despite lacking the cofactors in the AlphaFold 2 modeling; the spatial arrangement of the FMN domain and CaM against the NOS heme domain is also reasonable, with the FMN subdomain aligning well with HDX MS and mutational results; the CaM binding position is also realistic.

We have developed a hybrid strategy to overcome the challenges of assigning crosslinks in the homodimeric nNOS construct (69). By leveraging AlphaFold 2 predictions, we modeled distances between crosslinked residue pairs, an emerging approach for identifying inter-subunit crosslinks (82). Specifically, in a homodimeric protein (with subunits/chains A and B), where a crosslink between two residues produces four possible pairs (A-A, B-B, A-B, B-A), the pair associated with the shortest predicted distance relative to the structural model is prioritized as the most likely assignment. This approach, combined with visual mapping of the shortest crosslinks onto structural models, allowed us to identify inter-subunit contacts within the homodimeric nNOS-CaM complex (69). In parallel, we established ratiometric thresholds to distinguish crosslinks that preferentially appear in dimeric *versus* monomeric crosslinked species. This hybrid strategy not only reveals structurally plausible inter-subunit distance restraints but also integrates longer-range crosslinks that reflect the accessible conformational range. The identified inter-subunit NOS-NOS crosslinked residues, the lysines on the FMN subdomain (K765, K771) and the heme domain (K423, K452), are next to the putative conserved FMN-heme docking interaction sites (83); these were not detected in the CaM-free samples (Fig. 2). Our results clearly demonstrate CaM-driven formation of the output state (the CaM/FMN/heme docked complex). Several inter-subunit crosslinks additionally validate the topology of this complex, in particular in the context of CaM-heme and inter-subunit FMN-heme docking interfaces (Fig. 2).

**Figure 2:**
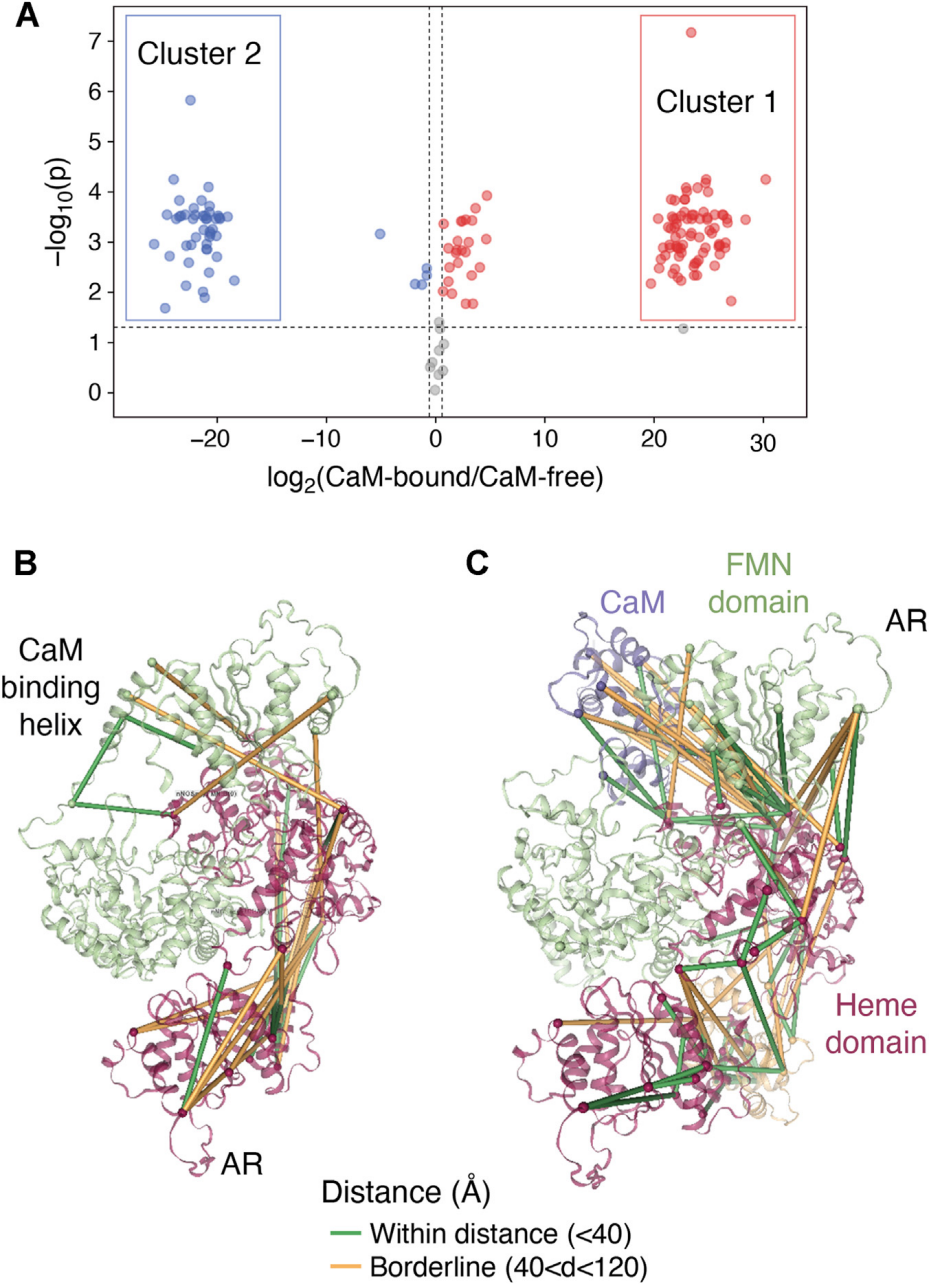
**Impact of CaM-NOS association on cross-linking patterns in homodimeric nNOS oxyFMN protein.***A*, Volcano plot showing the effect of CaM-NOS association on cross-link abundances of rat nNOS oxyFMN protein. Data were analyzed using a Benjamini-Hochberg adjusted *p*-value of 0.05 and a fold change threshold of 1.5. *Gray* cross-links indicate non-significant results (*p* > 0.05) or fold change <1.5. Outliers on the *left* and *right* (Clusters 1 and 2) represent cross-links unique to one sample type, with zero XL abundance values treated as 1 for inclusion in the ratio plot. Quantitation is based on the average of three replicate PRM experiments. Cross-links in the two clusters are mapped onto the top AlphaFold structural model, with distances ≤40 Å in *green* and >40 Å in *orange* (*panels B* and *C*, respectively). The colored domains and CaM protein are labeled with matching colored texts. Adapted from ref. (69).

All the unique crosslinks were then pooled into the PRM inclusion list for targeted LC-MS runs, and the peptide intensities were normalized in each run. Such PRM-based qXL MS was used for a comparative analysis of selected crosslinks across multiple samples under different conditions, *e.g.*, NOS alone vs. CaM bound, varying ionic strengths. The qXL MS results collectively demonstrate site-specific changes at the intersubunit docking interface and the CaM-responsive control element (the AR insert in the nNOS FMN domain) in response to CaM binding and ionic strength variation. The efficiency of intersubunit crosslinking serves as a measure of CaM-induced formation of the docked FMN/heme domains in the output state. CaM binding is the primary driver of remodeling the conformational landscape, while ionic strength further fine-tunes the conformational dynamics in the regulatory regions, particularly involving intersubunit FMN-heme docking interactions and the AR insert. This modulation of protein dynamics by CaM and ionic strength likely underlies variations in ET efficiency and its regulation. Our combined experimental approach using XL MS and AlphaFold 2 computational modeling (69) thus provides a sensitive measure of functional protein dynamics in the homodimeric nNOS oxyFMN protein.

Given that we mapped crosslinks onto the top AlphaFold 2-derived models, representing only a subset of possible conformations, we further utilized HADDOCK (84) to generate alternative rotations of the FMN subdomain that may reduce violations of the distance restraint. Rotational fit is the biggest unknown in the EM and HDX MS data. Notably, our HADDOCK models exhibit one fewer violation (69). However, the AR crosslinked pairs still exceed the distance restraint. This outcome is expected, as the AR insert is disordered, and the HADDOCK simulation cannot constrain the peptide fragment or induce greater order in the AR insert.

Various AlphaFold 2 models may provide putative snapshots of the inter-domain motions necessary for the ET. To create diverse AlphaFold 2 models (*e.g.*, undocked states besides docked states), shallow multiple sequence alignment (MSA), and reducing recycle numbers were recently applied for other proteins (85, 86). We have initially obtained and selected other distinct conformations for dimeric NOS oxyFMN with this MSA subsampling approach (69), in which the FMN subdomain is separate from the heme domain while still following its trajectory toward docking, *i.e.*, an in-transit state. Also note that the predicted template modeling scores of the selected alternative conformations are between 0.6 and 0.7, indicating moderate confidence in the relative positions of the domains. Importantly, combining the crosslink mapping results for the docked state with the ‘in-transit’ open/undocked states actually reduces the number of violations of distance restraints. This is expected, as the nNOS protein exists in an equilibrium of these conformational states, and the crosslinking reaction captures snapshots of these structural features under native conditions.

#### Rigorous design for XL MS workflows

To ensure the validity and rigor of XL MS data, a multi-pronged approach should be implemented to minimize potential artifacts (87, 88, 89, 90). First, performing both biological and technical replicates is critical for ensuring consistency in the data, as validating crosslinking patterns across multiple runs enhances reproducibility and minimizes the impact of random artifacts on interpretation. Second, a stringent false discovery rate threshold should be set, for example, at 1%, to further filter out unreliable identifications. Third, strict scoring filters within XL identification software, such as XlinkX and ΔXlinkX scores > 50 in Proteome Discoverer, should be employed to identify true positive crosslinks more confidently from background noise. Finally, DSBU crosslinks lysine and less frequently serine, threonine, and tyrosine residues. To tackle potential non-specific DSBU cross-linking (91), STY crosslinks should also be assessed. In our data, these typically comprised <10% of total KSTY crosslinks but still were included in the PRM quantitation for more comprehensive validation (69).

#### Applicability of XL MS to studies of protein dynamics in other systems

qXL MS also elucidates domain rearrangements and captures conformational changes in other protein systems under different conditions (92, 93) and disease states (94). Its application to multidomain protein systems remain nascent, with examples including transcription factors (93), chaperone proteins (95) and phycobilisomes (96). Beyond interaction mapping and conformational analysis, qXL MS has also expanded to explore diverse aspects of protein function, such as protein activation mechanisms and binding affinities in protein complexes (95) and protein-ligand interactions (97).

In recent years, XL MS has become a cornerstone to integrative structural modeling (98). Combining crosslinking data with deep-learning-driven protein modeling promises significant improvements in the resolution and depth of protein dynamics analysis (99). Innovations like photoactivatable (100, 101) and *in situ* crosslinking (68) methods, further offers the potential to capture protein conformational dynamics within native cellular environments.

Beyond structural analysis, qXL MS has also been initially applied to study changes in protein interactions (interactomes) in cells and organelles (102, 103). Notably, it revealed distinct interactome differences among various human cell lines (HEK293, HeLa, and MCF7), highlighting functional variations in processes such as chromatin remodeling and mitochondrial transport (104). These findings demonstrate the utility and reproducibility of qXL MS as a powerful tool for bridging protein structure, function, and cellular dynamics.

### Exploring conformations and dynamics in the NOS docked states *via* site-specific IR spectroscopy

#### Site-specific transparent window IR spectroscopy to characterize functional biophysics

Most existing studies of the NOS protein dynamics have focused on large-scale conformational changes involving the association of domains. The smaller-scale dynamics among substates within the docked state can also be important in ensuring efficient ET between domains. As is characteristic of interprotein ET, the tethered FMN domain is thought to approach its ET partner to first form an encounter complex, a loosely associated ensemble of states that includes a range of domain-domain orientations (105). This step is presumably followed by a rapid exchange with more well-defined states that are better suited for ET, while subtle conformational adjustments likely continue in the docked states (15). Emerging evidence further supports the importance of dynamic rearrangements within the docked states (15). Moreover, the partnering modules/domains form transient complexes (106) to balance between rapid ET and efficient turnover of the enzyme. Whereas the conformational sampling model (3) generally describes the association of the FMN and heme domains, the exact nature of their docked complexes remains under question and, furthermore, likely varies among the NOS isoforms (22). Therefore, a comprehensive understanding of the NOS mechanism remains incomplete without quantitative insights into the docked states.

A complete account of the docked states must include the population of an ensemble of substates (*i.e.*, conformational heterogeneity) and their rates of interconversion (*i.e.*, dynamics). Within the docked state ensemble, the structural differences among substates are small in scale. For example, structural details such as rearrangements of side chains could impact ET pathways between proteins. Such local interactions and dynamics are important for stabilizing the domain-domain contacts and mediating ET between domains. Interconversion between these substates could be rapid (ps-ns). However, capturing such dynamic states with high spatial detail is experimentally challenging. NMR relaxation measurements provide spectral densities of ps-ns dynamics, but slower multiple timescale dynamics and protein rotation complicate NMR analysis for a complex multidomain protein like NOS.

To address these challenges, IR spectroscopy emerges as a powerful method for studying protein dynamics due to its inherently fast timescale, which allows for the capture of rapidly interconverting states (107, 108). However, this technique is not without limitations. Spectral congestion prevents selective investigation of native vibrations, which poses a significant hurdle for detailed analysis. The problem can be overcome by the site-specific introduction of small amino acid side chains that have vibrational groups with 1900 to 2500 cm^-1^ frequencies, a “transparent” region free of intrinsic protein absorptions (Fig. 3*A*) (109). Basically, a small IR probe with a spectrally resolved frequency (*e.g.*, CO, cyano groups, azido groups, and carbon deuterium bonds) can be site-specifically incorporated into a protein, providing a spectrally isolated signal that is sensitive to the nature of the surrounding environment (109). The small size of the IR probes provides spatially localized information, allowing for high spatial resolution. The frequencies, number, and line widths of the IR absorbance bands in 1D IR provide a measure of the nature, number, and local heterogeneity, respectively, of the local environments. This transparent window region has traditionally been used to study hemeproteins by analyzing the vibration of small diatomic ligands (*e.g.*, CO) bound to the heme (110). In addition, any site within a protein can, in principle, be probed by introducing frequency-resolved labels at specific protein side chains (111, 112). Boxer pioneered this approach by developing vibrational Stark spectroscopy for proteins, initially using cyano (*CN*) group and other small molecule ligands, as highlighted in an early review of his work (113). Boxer later advanced the technique by incorporating *CN* at cysteine side chains (114). These elegant studies demonstrate the wealth of structural and dynamic information that can be obtained through *CN* labeling (115). FT-IR spectroscopy of transparent window probes has been widely applied for studying enzyme catalysis, protein folding, and PPIs (109, 116, 117).

**Figure 3:**
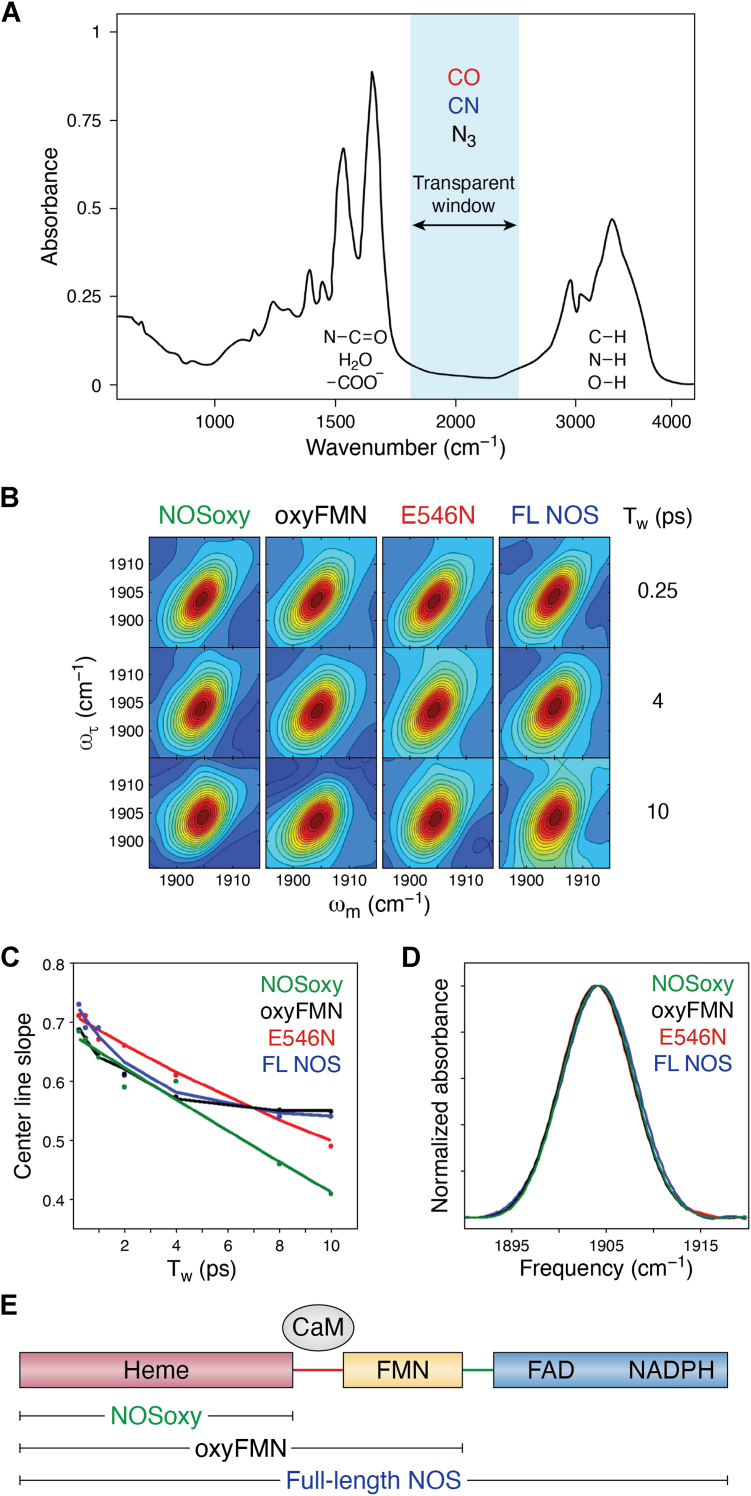
**Site-specific IR spectroscopy for probing protein conformations and dynamics: A case study on NOS.***A*, site-specific IR spectroscopy for elucidating protein conformations and dynamics. Specific sites of a protein can be characterized by introducing IR labels that absorb within the "transparent window" of the protein spectrum (approximately 1800–2300 cm⁻^1^), a range where native protein vibrations do not absorb. For example, Fe^II^−CO absorption can be utilized to probe the local environment of the heme center, while IR absorption of a cyano-labeled side chain enables targeted analysis of a specific residue site. These probes have IR absorptions in the transparent frequency window of a protein IR spectrum, thereby enabling the characterization of specific local environments and dynamics in proteins. *B*, representative 2D IR spectra at *T*_w_ of 0.25, 4 and 10 ps for Fe^II^−CO forms of NOSoxy, wt oxyFMN, E546N mutant oxyFMN, and full-length (FL) iNOS proteins. *C*, *center line* slope decay curves for these iNOS protein samples. *D*, 1D FT-IR spectra of the iNOS proteins. *E*, schematic of the NOS constructs.

While 1D IR offers significant advantages, advanced 2D IR techniques provide additional information that cannot be obtained with 1D IR alone (108, 118). The stronger dependence on transition dipole strength leads to the accentuation of bands to assist in spectral deconvolution. Moreover, 2D IR approaches enable rigorous measurement of inhomogeneous broadening, a measure of frequency heterogeneity, and allow for the determination of the timescale of the interconversion among the frequency distribution based on spectral diffusion, providing a measure of the heterogeneity of states and dynamics among them. Researchers can leverage the picosecond timescale of IR spectroscopy and the high spatial resolution of small IR chromophores to characterize localized environments along docking interfaces and measure interconversion between docked states. By combining advanced 2D IR techniques with site-specific placement of sensitive vibrational probes, this approach enables detailed elucidation of protein conformations and dynamics with high spatial and temporal resolution, proving invaluable in studying protein dynamics.

The evolution of site-specific 2D IR studies highlights its growing versatility and impact on protein research. Site-specific 2D IR studies of proteins initially focused on a CO ligand in myoglobin to measure side chain motions in the distal pocket (119, 120). Subsequently, CO and other small molecule ligands have provided insights into the dynamics within the active sites of cytochrome P450 (121), NOS (33), hydrogenases (122), and other enzymes (123, 124). The application of 2D IR spectroscopy has since expanded to transparent IR probes introduced at specific side chains to investigate protein folding, PPIs, surface hydration, and local rigidity (125, 126, 127, 128).

In the following section, we will present two examples of how transparent window IR spectroscopy has been applied to study domain-domain docking in NOS proteins, further illustrating its utility in understanding protein dynamics.

#### Protein dynamics in iNOS proteins using 2D IR spectroscopy

For our initial application of this approach to study NOS conformational dynamics, we directed our attention to the vibration of a CO ligand to the heme to assess the dynamics of the oxygenase domain. A set of time-dependent 2D IR spectra was obtained for the Fe^II^−CO complex of iNOS proteins (33). To evaluate the effect of a properly aligned FMN domain and bound CaM in the wild-type (wt) proteins on the IR spectra of the NOS heme site, two controls were used (Fig. 3*E*): the NOS oxygenase (NOSoxy) construct containing only the heme domain, and the E546N oxyFMN mutant with disturbed FMN-heme interdomain docking (129). These negative controls allow for comparison with the wt proteins, where the FMN and heme domains are properly aligned.

The oxyFMN construct contains only the oxygenase and FMN domains along with the CaM binding linker (Fig. 3*E*) (130). This is to preclude FAD/FMN interactions favor interactions between the FMN-binding domain and the oxygenase domain (131), facilitating study of the FMN/heme docked states. Since the interdomain FMN-heme contacts are specific (132), the docked IET complex structure should be similar to the oxyFMN and full-length NOS. Indeed, the docked FMN-heme complex models (13) were fit without any alteration into the EM density of the full-length NOS (11). The results for the oxyFMN construct should therefore apply to the full-length NOS.

The NOS protein sample could become an inactive P420 form under the laser beam during 2D IR experiments. In its ferrous state, NOS forms a Fe^II^–CO complex with a Soret absorbance peak at 446 nm (133), indicating retention of cysteine thiolate coordination to the iron center. However, protonation or disruption of the thiolate ligand, often due to denaturation or environmental changes, leads to conversion to the P420 form. This inactive state is characterized by a Fe^II^–CO complex with a 420 nm absorbance peak (134, 135). P420 conversion was observed during our initial 2D IR runs. This issue has been mitigated by adding H_4_B and by minimizing the amount of dithionite added for iNOS protein reduction. With these adjustments, high-quality 2D IR spectra have been obtained (Fig. 3*B*) without evidence of P420 formation (33).

2D IR spectra of wt oxyFMN and full-length iNOS proteins are similar, while the NOSoxy and E546N mutant show different spectra (Fig. 3*B*) (33). Moreover, different dynamics between the wt and control samples have been clearly observed (Fig. 3*C*). The results indicate that protein motions at the active site of the heme domain of the constructs containing the FMN domain or both the FMN and FNR domains are more restricted than those in the E546N mutant and the heme domain alone, due to the proper docking/binding of the FMN domain and CaM in the wt proteins. In other words, the local dynamics at the heme active site depend on having additional domains beyond the heme domain, and larger-scale dynamics influence the small-scale dynamics in the docked state. On the other hand, the 1D FT-IR spectra are nearly the same—the center frequencies and linewidths of the absorption bands of the iNOS proteins deviate by at most 0.1 cm^−1^ (Fig. 3*D*). This clearly demonstrates the additional insight provided by 2D IR methods that allow for a more rigorous measurement of local heterogeneity. Despite its limitation in location, the CO is a sensitive reporter of active site states facilitated by heme domain interactions with the FMN domain (33).

Considering that redox centers in proteins are sensitive to interactions that fluctuate on timescales ranging from slow motions of the global structure to rapid solvent dynamics, the timescales of motions potentially underlying the reorganization energy of ET between proteins are broad. Whether motions contribute to the reorganization of energy depends on whether the timescale is fast relative to the ET reaction (136). Consequently, rigidification of the protein environment of a redox center affords a way to minimize the reorganization energy of biological ET. A well-known example is the primary charge separation in photosynthetic reaction centers, where the protein response is essentially static on the timescale of ET (137). In the case of interprotein and/or interdomain ET, protein-protein and/or domain-domain association can result in overall slower fluctuations through the displacement of mobile water molecules at the protein and/or domain surfaces, restriction of surface side chain motion, and potentially the propagation of changes farther from the surface. Such changes are consistent with the slower inhomogeneous dynamics measured for the heme CO ligand in the iNOS constructs capable of forming the docked state. Unfortunately, 2D IR spectroscopy is not able to capture the timescale of the slow component of dynamics. However, if the timescale of motion becomes slower than the ET reaction, the contribution of the motion to reorganization energy would be eliminated, and the reorganization energy would thereby decrease. Such minimization of reorganization energy through PPIs and/or domain-domain interactions provides a possible mechanism to increase specificity in biological ET. The contribution to the nuclear energy activation from the free energy differences in biological ET reactions are often small and do not greatly differentiate reactivity among the massive number of possible redox partners within the crowded environment of a cell. Due to the critical function of NOS to produce the signaling molecule NO, the additional control of ET by modulation of the reorganization energy through domain association would serve to optimize signaling fidelity by minimizing undesired side reactions.

One may ask whether the limitation in time resolution of 2D IR could be addressed by recording complementarily 1D linear spectra, for which a resolution is available on all time scales. A linear spectrum captures the entire ensemble at one time point, and a 1D IR spectrum could be taken at a series of time points, with no limitation in the time resolution or timespan of the experiment. However, for a protein system at equilibrium, a series of linear spectra taken as a function of time would show no time dependence. A pump-probe experiment that measures the response after strong IR excitation only detects the dynamics of the excited state/ground state bleach populations (the pump-probe data can be retrieved through the projection of the 2D IR data onto the probe axis). 2D IR spectroscopy can measure dynamics among states within an equilibrium ensemble because it can connect the vibrational transitions of specific oscillators in the ensemble at different times through multiple field-matter interactions, which is not possible *via* 1D spectroscopy.

#### Probing CaM-NOS interactions by site-specific transparent window IR spectroscopy

Complementary charged residues on the interdomain interface guide the FMN-heme domain-domain docking (20, 83), as well as the docking between the FAD and FMN domains (138), and other multidomain proteins in general. The interdomain FMN/heme interface is well understood (20, 83), but the docking site is not a very promising target for development of selective NOS modulator because it is highly conserved among the NOS isoforms (139). Equally important, the CaM-heme(NOS) docking is required for proper FMN/heme domain alignment (13). In contrast to the FMN/heme interface, the CaM/heme interface is isoform-specific (22), which remains to be fully explored. Notably, there is a strong need to develop new intervention strategies selectively targeting different NOS isoforms as clinical selective NOS inhibitors remain lacking. An understanding of conformational dynamics that is linked to NOS function may provide new opportunities for inhibitor design by targeting interfaces between the functional domain modules. The “interfacial inhibitor” concept is gaining prominence in drug discovery, and many of such drugs have been FDA-approved for other targets (140). As such, it is of considerable interest to further investigate the CaM/heme(NOS) docking interface, which is isoform-specific and solvent-exposed, making it a potentially attractive target for rational development of new, direct therapeutics.

To elucidate how CaM shapes the docked state conformations and dynamics, we combine advanced protein IR techniques with the placement of sensitive vibrational probe groups, such as 4-cyano-l-phenylalanine (*CN*F), at specific domain-domain interacting interface sites. While the CO probe is limited to the heme center where it directly binds, *CN*F can be used to characterize specific sites along the docking interface. The overall workflow for site-specific IR spectroscopic investigation involves four main stages (Fig. 4). This protocol generally applies to other proteins as well.

**Figure 4:**
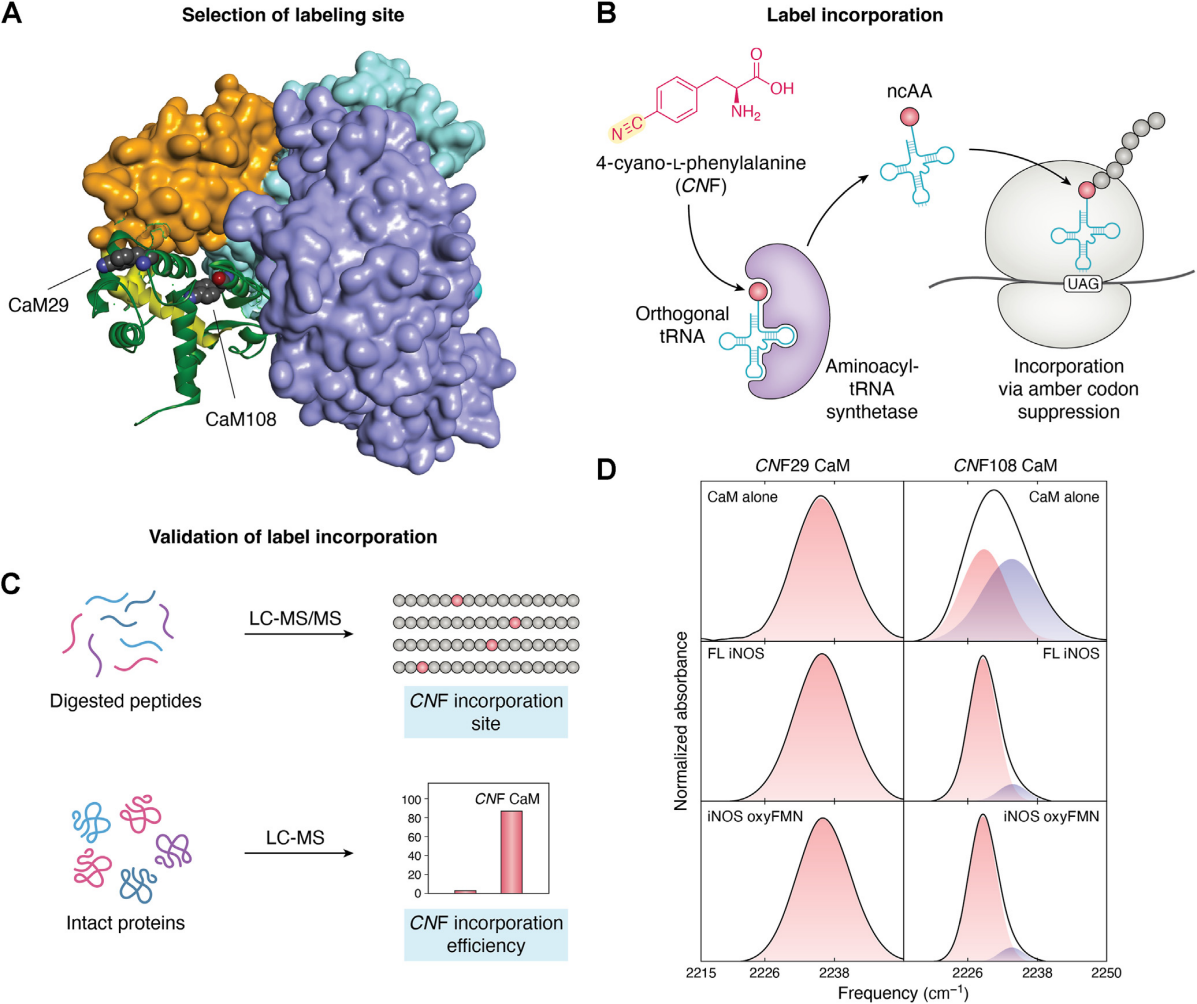
**The overall 4-stage workflow for site-specific IR spectroscopic investigations of the conformations and dynamics of the docked FMN/heme/CaM state in the NOS-CaM complex**. *A*, *CN*F is inserted at a selected, specific site along the docking region. For example, *CN*F can be placed at CaM 108 within the CaM-heme interface, serving as a sensitive vibrational probe for CaM-heme(NOS) docking. As a control, the *CN*F probe can be placed at residue 29, distal to any docking interface. CaM, shown as green ribbons, binds to the linker region (*yellow*) connecting the heme domain (*purple*) to the FMN domain (*orange*) in one subunit, while the heme domain in the other subunit, to which the FMN domain docks, is colored cyan. Additionally, CaM docks onto both the heme and FMN domains, with residue 108 at the CaM-heme(NOS) docking site. *B*, Amber codon suppression methodology is used to incorporate *CN*F, a ncAA, into the selected labeling site. *CN*F compound is added to the cell growth media, along with a specific tRNA synthetase orthogonal to the cell's natural translation machinery, typically introduced *via* a plasmid (*e.g.*, pUltraCNF). A second plasmid is used to insert the gene of the target protein (*e.g.*, CaM) containing a stop codon that incorporates *CN*F at the desired site. *C*, the specificity and efficiency of *CN*F incorporation into the purified recombinant protein are determined by LC-MS/MS of the digested protein and LC-MS of the intact protein, respectively. The enzymatic activity is also assessed to ensure minimal perturbation by the *CN*F label. *D*, the protein is then subject to IR spectroscopic measurements. Example FT-IR spectra of *CN*F29 CaM (*left panel*) and *CN*F108 CaM (*right panel*) are shown for the CaM alone (*top row*), as well as when associated with FL iNOS (*middle row*) or the iNOS oxyFMN construct (*bottom row*). The IR spectra are adapted from ref. (141).

First, the labeling sites in a protein are selected based on the mechanistic question and the structures or models of protein-protein complexes. For instance, site 108 in CaM is near the electrostatic CaM-heme(NOS) docking interface (Fig. 4*A*). Additional sites can be selected to enhance coverage, provide additional spatial information, and investigate other types of domain-domain interactions, such as hydrogen bonding and hydrophobic interactions. A distal site should be separately labeled as a control (see below). The site-specific IR label type also needs to be selected, mainly based on their IR spectroscopic properties and the feasibility of site-specific labeling; see below for detailed discussion.

Second, the IR label, typically a non-canonical amino acid (ncAA), is incorporated at the chosen site using a genetic code expansion approach (Fig. 4*B*). If the selected site does not permit ncAA incorporation, due to structural constraints or an unfavorable sequence context, the yield of the modified protein may be significantly reduced. Nearby sites may need to be screened to identify an optimal position that allows efficient ncAA incorporation without disrupting protein folding or function. This ensures high yields of stable, and functional protein with ncAA incorporation.

Third, site-specific incorporation and efficiency should be validated by mass spectrometry (Fig. 4*C*). Additionally, since the labeled protein is in fact a ‘mutant’ form, its enzymatic activities should be assessed to ensure that the variant does not significantly affect protein function and that the IR label serves as a ‘neutral’ reporter of the local environment at the labeling site/interface. Except for carbon deuterium labeling, the perturbation to protein function by introduction of any IR probe should be verified by appropriate assays. Among common probes, *CN*F is minimally perturbative as a Tyr or Phe analog, but the CN group can engage in non-native dipolar and hydrogen bonding interactions. Azides are larger but less prone to participate in strong noncovalent interactions, but see below for its limitations.

Fourth, the IR spectra of the protein sample are collected and analyzed. A set of samples should be examined to compare differences; representative FT-IR spectra are presented in Figure 4*D*. A native unlabeled protein sample is often needed to collect background IR spectra, which are essential for obtaining accurate spectra in the transparent window. Additionally, molecular dynamics (MD) simulations provide detailed information about the local environment around the IR labeling site. This includes interactions with surrounding residues and solvent molecules, which can influence IR spectral features. MD simulations bridge the gap between experimental IR data and the molecular-level details of protein structure and dynamics, enhancing the interpretation of IR spectra.

We will next present a case study of *CN*F labeled CaM in complexation with iNOS proteins (141) as an initial example of this workflow.

To fully realize the potential of this approach, it is essential to address key methodological considerations. Detection of absorptions of single vibrations in proteins, which are limited in concentration, is facilitated by IR probes with large extinction coefficients. In addition, site-selective incorporation of the IR probe is necessary. Among the site-specific IR probes (116), the aromatic C≡N group in *CN*F provides a relatively strong IR absorption (extinction coefficient in H_2_O, ∼200 M^-1^ cm^-1^) that can be accurately measured and characterized (142), and selective incorporation of *CN*F can be readily accomplished using an evolved tRNA/tRNA synthetase pair that incorporates a *CN*F at a specific position *via* orthogonal translation (143). *CN*F is preferrable to another popular IR probe, cyanocysteine, with regard to its selectivity: installing cyanocysteine at a single residue requires removal of all other native cysteines (144); the signals are also ∼ 5 times weaker for cyanocysteine than *CN*F. Cyanotryptophan is an alternative choice; tRNA/tRNA synthetase pairs for selective introduction *via* orthogonal translation were recently reported (145). Cyanoalanine is another option; however, the extinction coefficient of alkyl nitriles are weaker, and the labeled residues has to be inserted *via* semi-synthesis. A similar label, azidophenylalannine, gives a stronger IR signal than *CN*F and can be also site-specifically incorporated into a protein. Azidohomoalanine can be installed at methionine residues; the same challenge with labeling specificity in the presence of multiple methionines exists. However, a potential downside of azido probes is that Fermi resonances can complicate interpretation (146). Carbon deuterium bonds and alkynes also have absorptions in the transparent window, but their extinction coefficients are small.

Regarding the labeling site, *CN*F should be placed at a position that: (a) is anticipated to show a different degree of solvent exposure in Ca^2+^-CaM compared to Ca^2+^-CaM in complex with NOS, and (b) is adjacent to the CaM residues interacting with the isoform-specific NOS residues (22). For example, the docking model predicts that Val108 has a different degree of solvent exposure in the CaM-NOS complex and is at the side of the electrostatic patch that guides the docking (13). As such, Val108 should be sensitive to CaM-NOS complex formation, and the *CN*F108 CaM variant has been produced and examined. As a control, the *CN*F probe can be placed at CaM position 29, which is separate from the CaM-heme interface. Note that these two residues (108 and 29) are not *CN*F-labeled simultaneously in one protein. The residues are given here as an example of a specific labeling site and its control (Fig. 4*A*).

Co-expression of evolved tRNA/tRNA synthetases *via* pUltraCNF plasmid (143) can then be used to direct *CN*F incorporation in response to a TAG amber stop codon in a second plasmid containing the gene of the protein of interest (*e.g.*, CaM), enabling the incorporation of the *CN*F label at the specific site in CaM (Fig. 4*B*) (141). This second plasmid must be compatible with pUltraCNF, which carries the CloDF13 origin of replication (ori). Plasmids with the same ori are generally incompatible because they compete for the same replication machinery, leading to an unstable and unpredictable system. In the context of *CN*F incorporated CaM, the pET28a vector (147) can be used with pUltraCNF for incorporating *CN*F into the CaM protein alone. For co-expression of *CN*F CaM with iNOS, the pCWori vector is commonly used for NOS overexpression (141); in this case, the CaM gene is cloned into the same pCWori plasmid containing the iNOS gene to avoid the need for the host to maintain three plasmids and selection with more than two antibiotics, which could significantly inhibit *Escherichia coli* cell growth. Additionally, selection of *E. coli* strain is crucial to high-fidelity incorporation of ncAA, and C321.ΔA, a recoded *E. coli* strain lacking all amber codons and release factor 1 (148), generally outperforms standard *E. coli* competent cells in this regard. Initially, we did successfully generate milligram quantities of *CN*F-labeled CaM proteins with electrocompetent BL21(DE3) cells that have high transformation efficiency (≥1 × 10^10^ cfu/μg pUC19 DNA) whereas BL21(DE3) cells with a lower efficiency (10^9^ cfu/μg) failed to yield colonies. However, the transformation efficiency and overexpression performance of these high transformation efficiency cells often vary over time. Therefore C321.ΔA is preferred for more consistent production of *CN*F incorporated proteins.

After the *CN*F label incorporation, it is necessary to assess NO production activity to ensure that incorporation of the label does not disturb the CaM-NOS binding/docking. For example, *CNF*108 CaM activates rat nNOS enzyme with a NO synthesis rate of 61 ± 3 min^-1^ (80% of wt CaM-activated nNOS) (141).

Before performing any IR spectroscopic characterization, the site-specific incorporation of *CN*F must be confirmed by MS/MS analysis of the digested protein. Additionally, the *CN*F-incorporation efficiency can be determined using high-resolution intact protein MS. For instance, the observed mass of *CN*F108 CaM is 18,846.9 Da (141) after deconvolution analysis, matching the calculated mass of this CaM variant. For *CN*F CaM co-expressed with iNOS, intact protein MS is measured after the protein sample is pre-treated with formic acid in an organic solvent (*e.g.*, acetonitrile), which dissociates CaM from iNOS. Deconvolution analysis (149) typically yields mass peaks for both wt and *CN*F-labeled CaM, while truncated CaM forms or CaM with different residues at the incorporation site may also be detected. The incorporation efficiency can be inferred from the relative intensities of the MS peaks from these protein forms, as modifying a single site among 149 residues in CaM should not significantly affect the ionization efficiency of the intact protein. The LC-MS/MS and intact MS results (141) together confirm that the *CN*F probe has been effectively and selectively incorporated at the 108 position in CaM.

Notably, CaM is relatively small (16–18 kDa, depending on the presence of a His-tag or not), making it amendable to intact protein MS, even with basic MS instruments, to resolve the 25 Da mass difference from cyano modification. For larger proteins, such as cytochrome P450, *CN*F labeling efficiency has been often estimated by comparing the MALDI MS peak heights of unmodified and *CN*F-modified peptides from a digested protein sample (142). This approach assumes that *CN*F modification does not significantly alter ionization efficiency of the peptides or proteolytic cleavage near the *CN*F site. Although generally reasonable, this assumption could benefit from further validation, as the cyano group could impact on the electronic properties of the side chain, and the modification could be more significant for shorter peptide sequences (in comparison to the intact protein MS). An alternative is to add a known quantity of labeled protein as a calibration standard. However, uncertainties in protein concentration likely outweigh any variations in ionization efficiency. Another *CN*F% estimation approach involves comparing IR absorption intensities. Since the wt protein does not absorb in the relevant IR spectral region, its presence would lead to a decrease in the intensity of the *CN*F absorption band. By comparing the IR band intensities of the protein sample to those known variants, *CN*F incorporation at a specific site may be estimated (150).

FT-IR data indicate that the 29 site is unaffected by its association with the iNOS protein or peptide (Fig. 4*D*) (141). The insensitivity to *CN*F29 CaM’s association with the iNOS protein is anticipated given the 29 residue location, selected to be distant from the regions of interaction with iNOS. In contrast, the 108 site is sensitive to CaM–NOS complex formation: upon association of *CN*F108 CaM with iNOS holoprotein, the absorption envelope shifts toward lower frequency (Fig. 4*D*). IR spectra for *CN*F108 CaM are modeled as a superposition of two bands (Fig. 4*D*), indicating population of two major distinct states. Additionally, in the complex of *CN*F108 CaM with iNOS the linewidths are significantly (∼40%) narrower than *CN*F108 CaM alone (Fig. 4*D*). This suggests that the C≡N probe experiences a more restricted range of environments, reflecting side chain restriction in the complex with iNOS.

The C≡N frequencies are primarily influenced by electrostatic interactions (*i.e.*, Stark shifts) and hydrogen bonding, with hydrogen bonding being more temperature-sensitive (151). Temperature-dependent CN frequency analysis is useful for distinguishing between these mechanisms. Indeed, a notable difference between the 108 and 29 labeled CaM sites is their temperature-dependent behavior. For *CN*F29 in CaM, a single absorption band appears at 2235.8 cm⁻^1^, which matches the frequency observed for the free *CN*F compound in aqueous solution, indicating that residue 29 is indeed located on the solvent-exposed surface of CaM (Fig. 4*A*). Supporting this, the IR frequency decreases with increasing temperature (141). The observed frequency downshift for *CN*F29 with increasing temperature is consistent with the disruption of *CNF*29-water hydrogen bonding. In contrast, the temperature-induced spectral changes for *CN*F108 do not suggest hydrogen bonding involving its C≡N group. Instead, as the temperature increases, the *CN*F108 spectrum shifts upward and narrows, eventually appearing as a single band (141).

### How does phosphorylation modulate protein dynamics?

PTMs play central roles in cell signaling by dynamically altering the properties of a protein in response to changes in the cellular environment (152, 153). About 75% of all human proteins get phosphorylated. For example, on top of Ca^2+^/CaM being the primary means of activating NOS, phosphorylation further regulates NO biosynthesis *in vivo* (154, 155, 156, 157). Despite extensive efforts, the molecular mechanisms for the NOS regulation by phosphorylation remain enigmatic due to the challenges in obtaining a sufficient amount of phosphorylated NOS proteins required for detailed spectroscopic and kinetics studies. Typically, acquiring milligrams of phosphorylated protein is a daunting task, as *in vivo* phospho-protein levels are low, making purification from tissues impractical. *In vitro* phosphorylation by kinase could lead to unintended multiple phosphorylation sites and protein instability. The commonly used phosphomimetic approach, substituting a phosphorylated residue with a negatively charged aspartate or glutamate, has major limitations like pH charge disparities and structural differences. Furthermore, phospho-specific NOS antibodies do not recognize these substitutes, raising concerns about interpreting results from the phosphomimetic mutants.

To overcome these barriers, we have refined a recent synthetic biology method (148, 158) to site-specifically introduce phosphoserine (pSer) into rat nNOS holoenzymes (159, 160). We have investigated phosphorylation at a serine residue in the CT region (S1412 in rat nNOSα and S1446 in rat nNOSμ), which has well-established functional significance *in vivo* (161, 162). The general workflow preceding functional assays and detailed characterization is depicted in Figure 5*A*. Achieving efficient pSer incorporation into the NOS enzyme is a non-trivial task, as the orthogonal genetic expansion approach requires optimization for individual protein and remains an active area of research in chemical biology, and most work was done on smaller proteins like GFP. The generation of recombinant nNOS with pSer incorporation requires a toolkit consisting of a pSer orthogonal translational system (OTS), an expression plasmid encoding the nNOS protein, and a suitable bacterial host strain for protein production. We have optimized the phospho-nNOS preparation protocol using the Sep OTSλ system, the pCRT7 NT Topo vector, and the C321.ΔA ΔSerB strain for site-specific pSer incorporation directed by the TAG codon (148). Due to the potential instability of Sep OTSλ, it is essential to re-streak phosphoprotein expression strains from glycerol stocks onto fresh LB agar selection plates. This step serves multiple purposes, including assessing strain viability (*e.g.*, colony growth rate and uniformity) and identifying/excluding cells with mutant Sep OTSλ constructs, which often produce sporadic, larger colonies [23]. By adopting this practice, we have improved the reproducibility of recombinant phospho-nNOS production.

**Figure 5:**
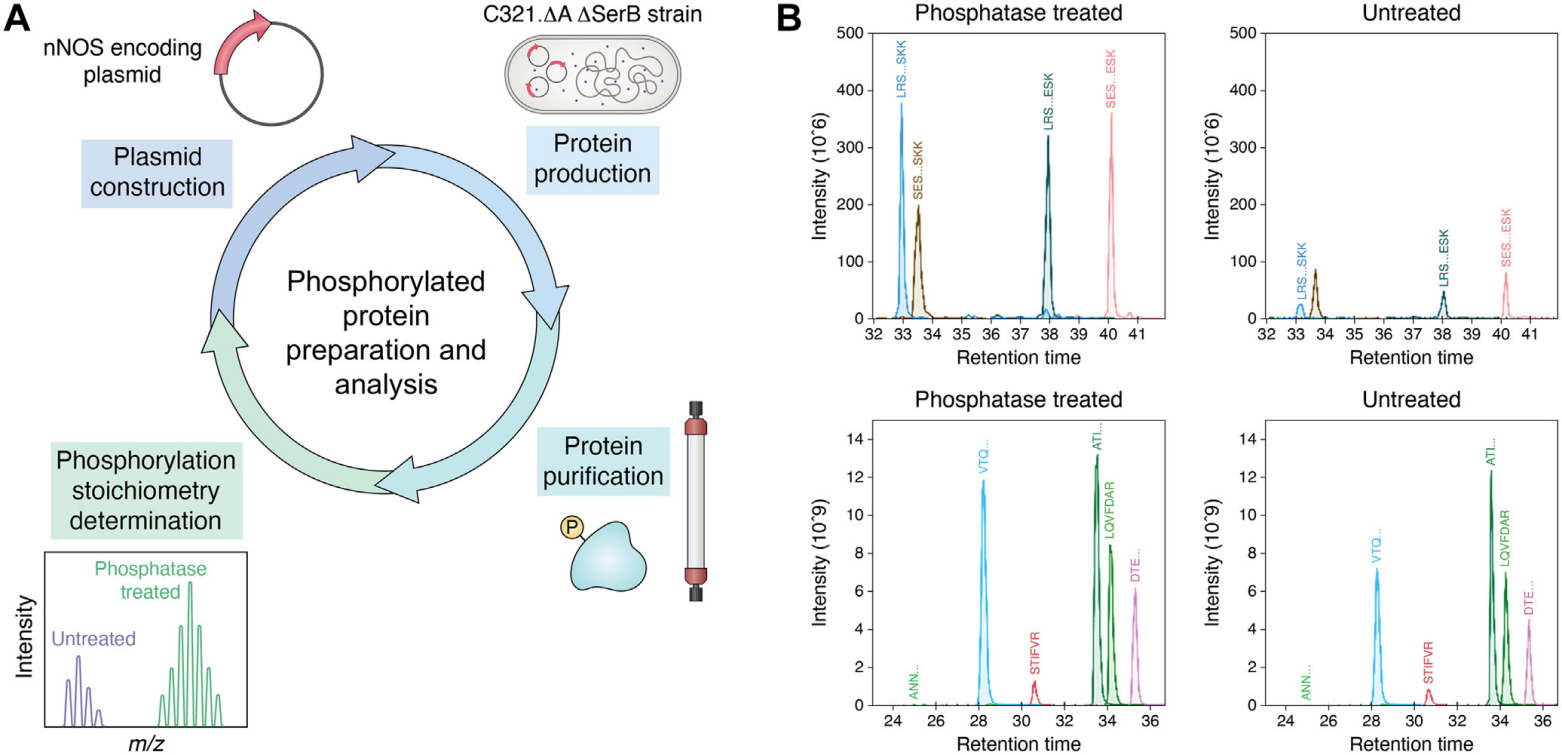
**Workflow for generation and characterization of genuine phosphorylated NOS proteins.***A*, overview of the workflow for preparation and analysis of phosphorylated NOS proteins prior to conducting functional assays and biophysical studies. *B*, representative LC-MS chromatogram of tryptic phosphorylated rat nNOS holoprotein, with and without phosphatase treatment, for phosphorylation stoichiometry determination. Adapted from ref. (165).

Additionally, it is necessary to ensure and validate high pSer-incorporation into NOS prior to any mechanistic studies. Determination of phosphorylation stoichiometry by LC-MS/MS is currently the method of choice by virtue of its high sensitivity and specificity. However, such MS measurements pose significant challenges (163) due to several factors, including different ionization efficiencies of phosphopeptides and poor MS/MS spectra of phosphopeptides. Moreover, impaired proteolytic cleavage near phosphorylation sites often results in the generation of multiple phosphorylated peptides, making absolute quantitation by MS costly and impractical. To address these challenges, we have tailored a label-free relative quantitation approach by PRM (164), where the pSer% quantitation relies on identifying and measuring the levels of each unphosphorylated counterpart peptide with and without phosphatase treatment (Fig. 5*B*) (159). Because only unphosphorylated peptides are measured by LC-MS/MS, the challenges related to impaired ionization efficiencies and typically poor MS/MS spectra of phosphopeptides are bypassed; the problem that arises from impaired proteolytic cleavages in proximity of phosphopeptides during protein digestion is also minimized. Importantly, several internal reference peptides were selected for normalization purposes, and their intensities were comparable in both treated and untreated samples (159). The similar observed intensities of the internal reference peptides demonstrate minimal sample loss during the sample preparation process (Fig. 5*B*), as both treated and untreated samples began with the same total nNOS protein amount. This tailored MS approach (159) is particularly significant, as large-size proteins pose challenges for intact protein MS, a conventional method typically used to measure ncAA incorporation in smaller proteins such as *CN*F labeled CaM (see above).

These tailored approaches now enable precise phosphorylation modifications of NOSs, opening a new line of research. For instance, through EPR spin trapping experiments, we have obtained direct evidence demonstrating the distinct effects of phosphorylation on O_2_^•-^ production by the nNOSα and nNOSμ variants (159). We speculate that phosphorylation at the CT site of nNOSμ may induce distinct allosteric conformational changes compared to nNOSα. These changes could explain the observed differences in O_2_^•-^ production. The notion of a pro-O_2_^•-^- conformation of NOS has been proposed in the literature for some time (165, 166, 167). However, proving or disproving this model presents a significant challenge. Currently, there is no straightforward method to directly monitor NOS conformations and link a specific conformation to O_2_^•-^ production. Furthermore, understanding of NOS conformations in solution is still limited, hindering comprehension of the structural mechanisms behind O_2_^•-^ production by NOS. Nevertheless, our initial study should inspire further detailed investigations into the factors influencing O_2_^•-^ generation from nNOS, such as heme reduction, O_2_^•-^ release, conformational equilibrium, and conformational states. The dynamic interplay between phosphorylation, conformational changes, and O_2_^•-^ production is a critical pathway for regulating protein functions in general, as well.

### Perspective

Conformational dynamics is as fundamental to biological function as structure. It is of current interest to understand the structural and functional basis of dynamic protein/domain interactions. A central question in multidomain enzyme catalysis concerns how functional domains collaborate and interact to dynamically respond to external cues, such as PPIs and/or PTMs, which introduce additional key layers of regulation to biological processes. Given the large number of variables to deal with, it is no surprise that this question has not been fully answered yet.

The chemistry of mammalian NOS, a redox enzyme consisting of multiple rigid domains connected by flexible linkers, is coupled with major dynamical rearrangements during catalysis. The functional interaction between the reductase domain and the heme domain is central to understanding the mechanism of IET and catalysis. Although high-resolution crystal structures of isolated NOS domains represent a significant step toward understanding the molecular function of NOS proteins, the vast flexibility and mobility of the domains makes crystallization of the holoenzyme particularly challenging and, to date, no structural data of any holo-NOS isoform has been possible. Moreover, static crystal structures cannot fully capture the dynamic conformations of proteins under native conditions, as they often represent a single, low-energy state in a crystalline environment. The all-important structural specifics of how CaM activates NOS thus remain elusive. The large size and dynamic nature of NOSs necessitates the use of integrative in-solution biophysical methods.

The technological revolution is driving radical advances in what is possible in protein science. These exciting advances include biophysical and spectroscopic techniques for studying protein conformations and dynamics in solutions, synthetic biology methodologies for generating phosphorylated proteins or incorporating spectroscopic labels (*e.g.*, IR) *via* site-specific incorporation of ncAAs, and AI-based computational modeling of proteins and their complexes. Furthermore, we envision that combining data from complementary methods (*e.g.*, XL MS, cryo-EM, HDX MS, pulsed EPR) will provide detailed information to correlate the domain dynamics with IET and enzymatic activity of the NOS isoforms. These techniques highlighted in Figure 6 have proven successful in studying NOSs on their own (10, 30, 34, 168). Looking ahead, an integrative approach will dramatically expand the scope and depth of the experimental measurements (16), offering a stronger foundation for computational modeling and ultimately yielding a more comprehensive understanding of the conformational dynamics that underlie function. Notably, high-resolution structural analysis of the NOS protein has been hampered so far by its highly dynamic and heterogeneous nature, making it an ideal target for investigation by a combination of low- and medium-resolution structural techniques. XL MS is ideal in this regard by providing distance relationships in large proteins (169, 170), and has initially revealed its power for studying the NOS-CaM complex (32, 69). Overall, the envelope of the protein is to be reconstituted from EM images and used to guide further docking and modeling studies by combining the XL MS and HDX MS data (84), while also considering known structure-activity relationships. Such integrative approach aims to provide high-confidence structural models and conformations of the large modular NOS enzyme and its complex. The models are expected to predict conformational changes upon the functional modifications (*e.g.*, PPIs, PTMs and ligand binding). The plausible model/mechanism is testable by kinetics and spectroscopic studies of mutants at the predicted interacting sites (22). For instance, XL MS will be leveraged to map and identify domain-domain interaction regions, and site-specific IR spectroscopy will be employed to further probe these interfaces in detail. This is clearly a fertile area for future study.

**Figure 6:**
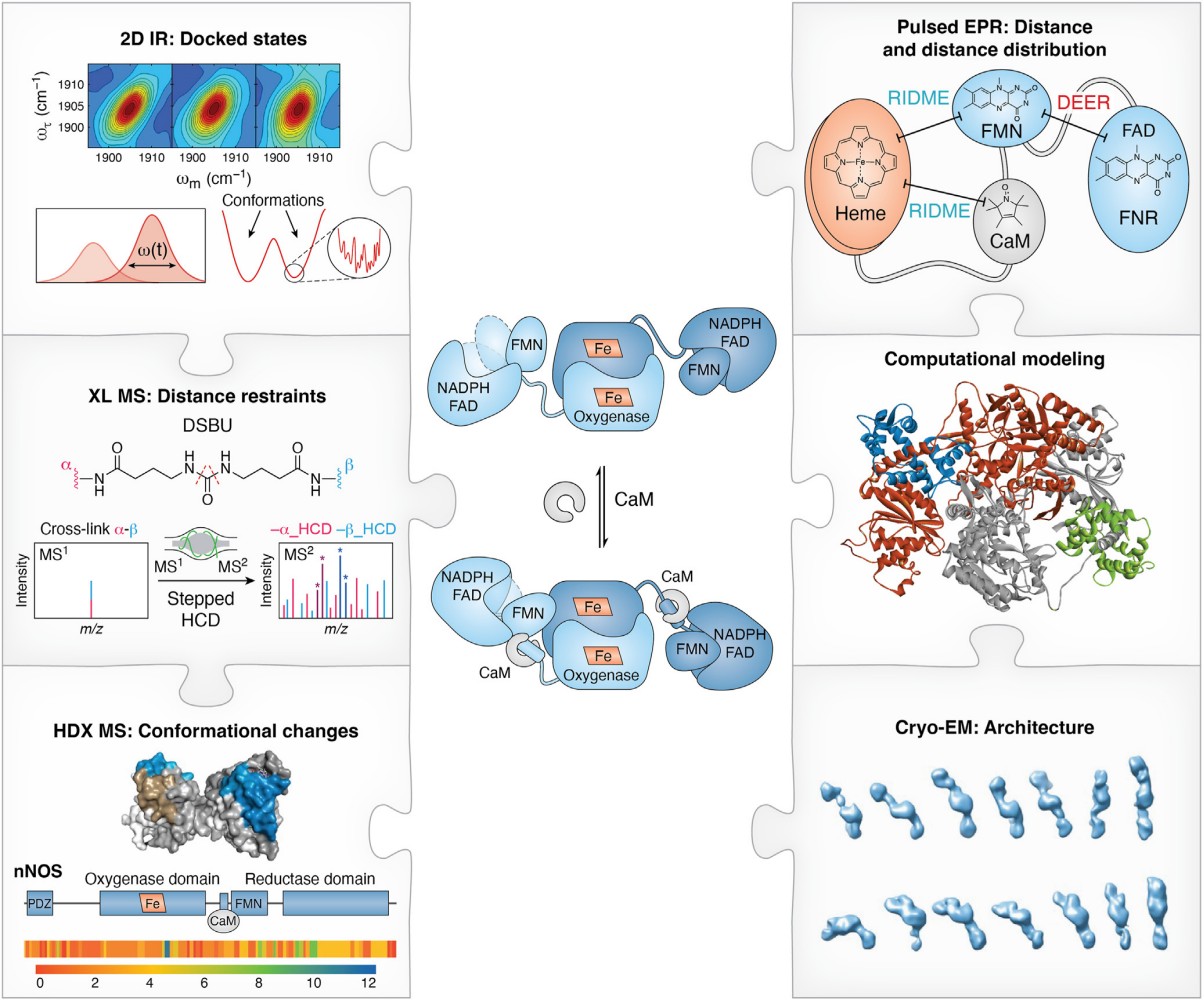
**Dissecting the functional protein dynamics of NOSs (center) through an integrative research program.** By combining in-solution experimental restraints with computational modeling of structures and conformations, this approach presents a promising strategy to build high-confidence models of higher-order structures/architectures and disentangle conformational distributions, shedding light on the regulatory mechanisms of NOS enzymes. The experimental tools presented here serve as examples, while other advanced techniques, such as cryo-TM, single-molecule FRET, and NMR, can be integrated into the workflow to further enrich our understanding of the conformational landscapes of multidomain proteins in general.

Although considerable progress has been achieved in recent years, some important questions and methodological/workflow issues remain to be addressed and certainly merit further investigations and refinements. These are summarized as follows.

(1) Site-specific IR approach enables (a) analyzing local microenvironments at the protein docking sites, and (b) determining equilibrium dynamics, *i.e.*, interconversion rate between the docked states (171). Beyond labeling the CaM 108 site, other CaM and NOS residues along the domain-domain docking interfaces should be labeled to increase coverage and provide additional spatial information. In addition to *CN*F-incorporated CaM, labeling the NOS domain-domain interfaces will complement the *CN*F CaM study and enable a more detailed investigation of the FMN-heme and CaM-NOS interfaces. The three NOS isoforms (wt and mutants), which vary in ET rates (172) and turnover number (46), are of particular interest for site-specific *CN*F labeling. Comparing the conformations and dynamics by IR is expected to evaluate whether (and how) the docked state is involved in their different bulk FMN−heme IET rates, based on a conformational dynamics model (15). The foundation of transparent window 2D IR has been laid, along with the site-specific labeling, and the approach is now primed for expanded applications to deepen the understanding of functional protein dynamics (125). Other IR probes, such as cyano-selenocysteine with longer lifetime, could be considered to extend 2D IR's accessible dynamics, tackling signal decay linked to probe lifetimes that limit the dynamic range (125).
(2) Targeted qXL MS enables comparative analyses of selected crosslinks across multiple samples at different functionally relevant conditions (*e.g.*, CaM and/or Hsp90 binding, phosphorylation, NADP^+^ ligand binding). A robust test for interpreting these data involves comparing mutants that inhibit FMN-heme docking (20), which disrupts inter-subunit crosslinks. This comparative qXL MS approach strives to address long-standing questions: how does the regulatory element search the conformational space, and how is this changed by CaM binding without blocking the domain-domain interactions? To shed light on the structural basis of the ET control, the relative positioning of the inter-subunit FMN-heme crosslinks and the NOS-unique control elements (42), should be carefully scrutinized. Besides DSBU, other crosslinking reagents (72) that differ in spacer lengths (173) and targeting residues (174) have been utilized to enhance the spatial resolution and coverage for other proteins. For instance, a heterobifunctional photoactivatable cross-linker, sulfo-SDA, was used to provide high-density data (174), aiding analysis of conformers of human serum albumin and cytochrome *c* proteins.
(3) Protein modeling methodologies, such as HADDOCK, frequently utilize XL MS-derived distance restraints and crosslinked residues to guide domain-domain docking in the pursuit of energy minimization. The individual input structures are from either crystallography or protein folding predictions, such as AlphaFold 2. However, X-ray crystal structure, commonly used to train deep learning-based structure prediction methods, may lack representation of structural flexibility, multiple conformations, and dynamic interactions. An exciting advancement is the introduction of AlphaLink (68) and AlphaLink 2 (175), which integrate crosslinking restraints into deep learning during training. Additional programs, such as ColabDock (176) and CombFold (177), have also emerged, leveraging crosslinking data to enhance the accuracy of protein structural predictions. It is intriguing to incorporate these innovative tools from such dynamic and rapidly evolving field into the integrative modeling workflow. Of note is that biasing the computational models with HDX MS restraints have lagged far behind, as HDX perturbation are much nuanced and multivariable than the simple straightforward distance restraint afforded by a crosslink.
(4) Conformational transitions were linked to eNOS S1179 phosphorylation-stimulated ET (178). However, they used a S1179D mimetic mutant. Utilizing actual phosphoserine offers much more physiologically relevant information than employing the traditional mutation approach. Intriguingly, a recent HDX MS work revealed CaM-induced conformational changes near the phosphorylation sites (36). Driven by these facts, it is valuable to leverage the pSer-incorporated NOSs to further investigate how phosphorylation remodels the functional dynamics using the integrative toolbox of protein IR, XL MS, rapid kinetics, and activity assays (Fig. 6). The effects of phosphorylation on functional dynamics may be reversed through phosphatase treatment (to dephosphorylate the protein), as a control. The importance of other PTMs, such as acetylation, has been increasingly recognized (156, 179). Emerging tools (180) can be utilized to site-specifically incorporate these ncAAs, which may also enable diverse applications including control of protein function.
(5) AlphaFold 2 is designed to predict a single, converged structure for a protein sequence. However, many proteins like NOSs adopt multiple conformations, especially in the presence of interacting partners or molecules, such as ligands and drugs, which are crucial to their function. Those multiple conformations are missing from AlphaFold 2 database (181). To diversify the models and generate multiple conformations, several strategies have been employed, including shallow MSA and/or reduced recycle number (85, 86), as well as random seeds (182), to predict conformational ensembles of various proteins. Another approach involves clustering the MSA (183, 184), generating several sub-sampled MSA files based on the minimum number of samples required for each cluster in DBSCAN; however, this AF-cluster method may result in slightly less accurate models due to reduced MSA depth, and hasn’t been validated for multimers. Additionally, *in silico* mutagenesis “SPEACH-AF” (185) has proven to be as effective as the other methods, and in some cases, may even yield better results depending on the specific protein system. Undocked states of dimeric NOS protein have been initially generated using AlphaFold 2 subsampling (69), and manual examination of the models against crystal structural and architectural data shows that the FMN subdomain is quite far from the heme domain, while it is still in the route to dock onto the inter-subunit heme domain. A caveat in the AlphaFold 2 prediction of conformational ensemble is that it cannot predict the energies of the structures, and their relative populations. Fortunately, the population and distribution information of NOS proteins can be measured by experimental tools such as fluorescence lifetime (15, 105) and pulsed EPR (28). Of note is that NMR has been employed in conjunction with XL MS data to explore alternative conformational states in other proteins (184, 186). It is intriguing to generate diverse conformations of NOS proteins, cluster the models, identify principal components in each cluster, and compare their densities with experimentally measured population values. These representative conformations may also be validated with crosslinking data. Moreover, combination of mapping results against each principal component is expected to enhance the coverage of crosslinked pairs within the crosslinker distance restraints. Notably, diffusion algorithms like AlphaFold 3 (187), released in May 2024, have been enhanced through training with AlphaFold-Multimer; these advancements now enable modeling of biomolecular complexes, including DNA, RNA, cofactors, and ions, along with residue modifications such as phosphorylation. AlphaFold three represents a significant advancement in structural modeling (188), especially for elucidating the intricate architecture of smaller or higher-affinity protein complexes (189). However, its lack of public availability means it has not been thoroughly assessed yet. Google DeepMind recently made the AlphaFold3 inference code and model weights accessible *via* a GitHub repository, albeit with significant restrictions on its use. Additionally, the increased complexity of multidomain protein systems has been highlighted by predictions that feature highly complementary structural interfaces and the positioning of flexible linkers near the core and periphery of generated structures (189). It is anticipated that AlphaFold three-inspired code, once the executable including algorithm model weights is released, will be developed by the community and shared to achieve performance equivalent to, or higher than, what has been reported (187). It has already been remarkable to see numerous reimplementation efforts emerge within months of AlphaFold 3’s release, even though many are still constrained by licensing issues. Excitingly, alternative software solutions, such as Boltz-1 (190) and Chai-1 (191), aim to replicate AlphaFold 3 with fewer restrictions. However, these alternatives still require comprehensive validation to confirm their reliability and effectiveness.
(6) Despite extensive experimental data on docking probabilities and dynamics for various NOS isoforms (9, 28, 38), a quantitative comparison or analysis of these results is not yet feasible due to the lack of a unifying theoretical or computational approach to quantitatively explain the experimental findings and rationalize any observed differences. This challenge is compounded by the fact that all conformational aspects are interrelated: the formation of the docked state for a given pair of modules (*e.g.*, NOS domains and/or CaM) depends on their interaction energy and available conformational space, which is, in turn, influenced by other modules. Conversely, the formation of the docked state in one module constrains the conformational space of other modules, affecting their dynamics and promoting the formation of additional docked states (30). The NOS field has reached a critical juncture, where the pursuit of elucidating the functional mechanism is hindered by the absence of comprehensive quantitative knowledge about the conformational behavior and its impact on IET processes. Addressing this fundamental problem requires a convergence of computational analysis and experimental approaches, where experiments refine computational models, and computational insights help interpret experimental results. The synergistic nature of this combined approach will enable the determination of three, key conformational properties/aspects: statistical distributions, energy landscapes, and dynamics. This will allow for a unified explanation of diverse experimental data and rationalization of IET rates based on the inherent NOS molecular architecture. A recent study combining pulsed EPR and modeling (192) represents an important step toward a more comprehensive understanding of the roles of major conformational and intrinsic tunneling components in the FMN−heme IET step during NOS catalysis.

### Final remarks

Multidomain proteins represent a substantial portion of the human proteome, yet their dynamic behavior remain challenging to study. These dynamics are crucial for regulatory processes and are often linked to disease states. Understanding the domain movements and local domain-domain docking dynamics within these proteins is a frontier challenge, particularly for elucidating ET and signaling mechanisms. Fortunately, recent advancements in AlphaFold 2 and other AI-based modeling techniques have revolutionized our ability to predict protein structures with remarkable accuracy. Additionaly, these methods now enable the generation of conformational ensembles that reflect the dynamic nature of multidomain proteins. Notably, AI-based modeling has become increasingly cost-effective, opening new doors for researchers. For instance, ColabFold can be easily installed on an affordable Linux OS (*e.g.*, Ubuntu) workstation, enabling swift predictions, such as those for dimeric NOS proteins with over 3000 amino acids, completed in just a few hours using a 48 GB GPU. Many academic clusters also support AlphaFold jobs, making them readily accessible *via* SSH in a Windows environment. Python scripting further enhances flexibility, enabling customizations like subsampling with random seeds. With its low cost, ease of use, and adaptability, AI-based modeling is rapidly becoming a transformative and accessible tool for routine research, with adoption among researchers steadily increasing.

To achieve the necessary spatiotemporal resolution and address fundamental questions, integrated approaches are essential. These approaches collectively aim to deliver quantitative data on key aspects including docked state conformations and dynamics, elucidate structural changes tied to enzymatic outcomes, and provide deeper insights into the regulatory inputs, such as phosphorylations. Given that NOS is a paradigm signaling system, the results of such studies will have broad-reaching impacts.

One major challenge has been the design of selective NOS inhibitors, due to the highly conserved heme active sites across the three human NOS isoforms. A deeper understanding of functional protein dynamics could facilitate rational design of selective modulators for these isoforms. Moreover, this research holds significant mechanistic implications for a broader family of modular enzymes, including P450 reductase and sulfite oxidizing enzymes. The methods developed for studying NOS proteins as a model system provide valuable opportunities to advance investigation into the conformational characteristics of other multidomain enzymes in general. This is an exciting frontier, rich with potential for groundbreaking discoveries.

## Data availability

All data are contained in the manuscript.

## Conflict of interest

The authors declare that they have no conflicts of interest with the contents of this article.
